# Tissue engineering is a promising method for the repair of spinal cord injuries (Review)

**DOI:** 10.3892/etm.2013.1454

**Published:** 2013-12-18

**Authors:** WENCHEN JI, SHOUYE HU, JIAO ZHOU, GANG WANG, KUNZHENG WANG, YUELIN ZHANG

**Affiliations:** 1 Medical School of Xi’an Jiaotong University, Xi’an, Shaanxi 710061, P.R. China; 2 Department of Physiology, College of Medicine, University of Sydney, Sydney 2006, Australia; 3 Department of Surgery, The Third Affiliated Hospital, Medical School of Xi’an Jiaotong University, Xi’an, Shaanxi 710068, P.R. China

**Keywords:** tissue engineering technology, spinal cord injury

## Abstract

Spinal cord injury (SCI) may lead to a devastating and permanent loss of neurological function, which may place a great economic burden on the family of the patient and society. Methods for reducing the death of neuronal cells, inhibiting immune and inflammatory reactions, and promoting the growth of axons in order to build up synapses with the target cells are the focus of current research. Target cells are located in the damaged spinal cord which create a connect with the scaffold. As tissue engineering technology is developed for use in a variety of different areas, particularly the biomedical field, a clear understanding of the mechanisms of tissue engineering is important. This review establishes how this technology may be used in basic experiments with regard to SCI and considers its potential future clinical use.

## 1. Introduction

Spinal cord injury (SCI) typically results in a permanent disability, which may be an economic burden on the family of the patient and society, and to date there is no effective method of treatment (1). Every year there are 12,000 new cases of SCI in the USA, with the total number of American individuals living with a SCI estimated at ~259,000 (2). The development of a cure for SCI is a research topic of particular interest.

SCI triggers a series of pathological steps that include the original insult and subsequent secondary steps, such as ischemia, anoxia, free-radical formation and excitotoxicity (3). The original insult refers to the mechanical trauma that leads to the SCI. During this period, the spinal cord tissue is disrupted by an external force, produced by the original insult mechanism. The most recognized mechanism for injury includes the following steps (4,5): i) contusion of the spinal cord when injury occurs and ii) prolonged compression due to displacement of vertebral bony structures and tissues. Following the initial spinal cord trauma, secondary damage is apparent. The post-trauma inflammatory response is particularly significant and, through a series of complicated cellular and molecular interactions, plays a key role in the entire secondary phase following SCI (6,7).

Clinically, the treatment of SCI mainly focuses on reducing secondary damage and the prevention of complications (8). However, if the aim is to successfully repair the SCI and promote functional recovery, the following must be achieved (9,10): i) reduction of the death of neuronal cells, ii) inhibition of glial scar formation, since glial scarring decreases axon growth, iii) provision of a matrix at the injury site to supply the nutrients required to support axonal growth, iv) elimination of immune reactions and v) facilitation of the build-up of functional synapses and the transmission of neurotransmitters by regenerating axons. The key to treatment is to establish axonal regeneration in the damaged area with the anticipation that it will extend through the damaged area to establish a connection with the target neurons in the spinal cord. To date, it has been demonstrated that blocking inhibitory molecules and antagonizing secondary injury mechanisms promotes axon growth, by using trophic factors, cellular transplants and polymeric scaffolds (11). However, no significant functional recovery has been observed; therefore, a novel method is required.

**Figure 1:**
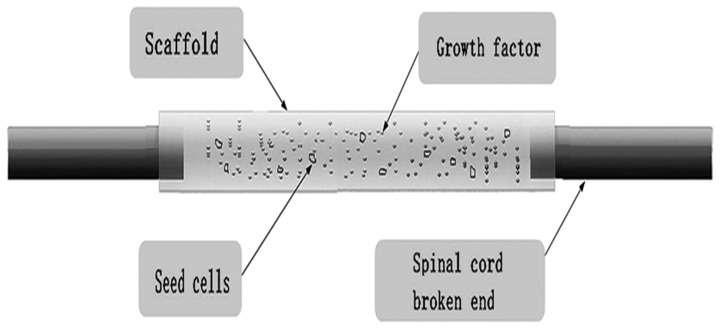
Scheme for the use tissue engineering technology in the repair of spinal cord injuries.

Tissue engineering is an emerging area in biomaterial research that possesses great therapeutic potential. However, in order for it to be used clinically there are challenges that need to be overcome (12). In recent years, studies have begun to explore the possibility of using tissue engineering technology to repair SCIs, specifically, by using seed cells, neurotropic factors and a biological scaffold (Fig. 1). The aim of this review is to discuss this tissue engineering method and investigate the hypothesis that, if a suitable seed cell, scaffold and growth factor are identified, tissue engineering offers bright prospects for future research and the potential for clinical use.

## 2. Seed cell

The basis for tissue engineering is the seed cell, which is also the bottleneck that has been restricting the development of tissue engineering. The main reason for this is that certain cells, such as cartilage cells and endothelial cells, are limited and it is not possible to construct a large organization through small quantities of tissues *in vitro* (13). In general, tissue engineering seed cells must meet the following criteria (14,15): i) successful ability to proliferation *in vitro*, ii) good cell viability and function, iii) high level of purity, iv) no rejection by the immune system and v) high safety. With the continuous development of tissue engineering, stem cells, including embryonic stem (ES) cells, and adult stem cells are gradually entering the seed cell field, with many advantages, including wide availability, a strong ability to proliferate, the ability to differentiate into a variety of cells and the ability to form the corresponding organization. At present there are clinical problems with using seed cells, the selection of a suitable seed cell may resolve this problem (16).

ES cells and neural stem cells are the most important types of stem cell that were used in the early stages of seed cell research (17,18). Transplanting ES cells into the brain was shown to significantly improve neurological function in an animal model of Parkinson’s disease (19). The reason for this success is that the cells survived and were differentiated into different cells, such as oligodendrocytes, astrocytes and neurons. However, this method has certain ethical issues and problems with regard to rejection reactions (20). In addition, the availability of neural stem cells is limited and, therefore, their widespread clinical use is not viable (21). With advances in stem cell research, mesenchymal stem cells (MSCs) extracted from bone marrow (BMSCs) have been shown to contain pluripotent precursor cells, which have the ability to differentiate into various types of brain cell (22). *In vivo* transplantation of BMSCs into the brain has established that they migrate throughout various brain regions where they undergo differentiation into cells with astrocytic and neuronal phenotypes (23). However, if BMSCs are to be used clinically, the extraction of bone marrow from patients is necessary, which is likely to result in patient trauma. Therefore, an increasing number of studies have suggested the use of adult stem cells, which have all the advantages of BMSCs, but without the need to induce trauma in the patients in order to extract them.

Muscle-derived stem cells (MPSCs) are adult stem cells, commonly used in tissue engineering. Alessandri *et al* (24), have demonstrated that adult human skeletal muscle includes a population of progenitor stem cells capable of generating cells of the same lineage and have suggested that MPSCs are ideal seed cells. Lavasani *et al* (25) and Wu *et al* (26) have shown that MPSCs have the ability to differentiate into various cell types when placed within specialized inducing media. Danisovic *et al* (27,28) focused on the immunological properties of MPSCs and hypothesized that their potential for differentiation may be useful in cell therapy for a variety of degenerative diseases. However, the majority of studies have investigated the use of MPSCs to cure coronary and urological diseases, with a limited number using MPSCs to repair nerve injuries. Woo *et al* (29) transplanted MDSCs into a cavernous nerve injury in a rat model, the result of which demonstrated that MDSCs were capable of improving erectile function. Stulpinas *et al* (30) and Shibuya *et al* (31) investigated the potential use of MPSCs in the treatment of non-acute myocardial infarction, with a swifter recovery. Kwon *et al* (32,33) found that although MPSCs are a good seed cell, capable of differentiating into numerous cell types, the neural differentiation capacity of MDSCs is less than that of adipose-derived stem cells (ADSCs). Therefore, ADSCs may be the most suitable type of adult stem cell for use as seed cells in SCI treatment.

Adipose tissue is abundant in the body. Zuk (34) extracted cells from adipose tissue and observed that the morphology, biological characteristics and immune phenotypes of the cells were similar to those of BMSCs. An advantage of using ADSCs is that obtaining these cells is minimally invasive to the patient. If different types of induction medium are used, the cells may differentiate into adipocytes, osteoblasts, chondrocytes and neurons, which are the most common types of seed cells currently used in research, and represent a promising tool for SCI treatment (35–38). Recently, studies have shown that due to the secretion of various growth factors, such as hepatocyte growth factor (HGF), tumor necrosis factor-α (TNF-α), vascular endothelial growth factor (VEGF), brain-derived neurotropic factor (BDNF) and nerve growth factor (NGF), ADSCs may be used in the acute stages of SCI and have the potential to improve functional recovery, tissue preservation and neuronal regeneration (39–41). Oh *et al* (42) used ADSCs to treat SCI and observed functional improvements. Similar results were demonstrated by Barriga *et al* (43), Ferrero-Gutierrez *et al* (44) and Chung *et al* (45). Therefore, this type of stem cell may be considered to be the most suitable for use as a seed cell for the treatment of SCI.

## 3. Scaffold

Maintaining cell growth is challenging if the cells are injected directly into the damaged area. Opening the meninges may lead to cell loss, which is likely to inhibit the ability of the cells to adhere to the damaged tissues and proliferate. Therefore, in addition to seed cells, tissue-engineering scaffolds are also important and their potential use in the repair of the spinal has been the subject of study for several years (46).

The requirements of a scaffold for spinal cord regeneration are as follows (47,48): i) good biocompatibility, in order to avoid reactions with the immune system, ii) an ideal degradation rate and the formation of non-toxic degradation products, and iii) mechanical properties that are suitable for cell adhesion and axonal regrowth. There have been several studies that have concentrated on the microstructural design of porous scaffolds, which must be conditioned *in vivo* prior to implantation (49,50). However, this has the major disadvantage of increasing the difficulty of the design at the engineering level and surgical implantation may also be challenging. The best solution is to create a scaffold with a simpler design, that is easier to transplant and is suitable for various types of injury (51,52).

Silk fibroin (SF) is obtained by degumming silk and acts as a natural structural albumen without physiological activity. It mainly consists of three simple amino acids: glycine, alanine and serine, which account for 85% of the total protein. Furthermore, SF has excellent mechanical properties, good compatibility and induces only a slight inflammation reaction *in vivo*. SF has been used as a scaffold for the treatment of SCI in certain experiments, but the disadvantage of this material is that when it is dry it is particularly brittle and difficult to handle (53). Therefore, in order to overcome this shortcoming, another polymer, chitosan, is added to the SF formulation. Chitosan has been investigated for its biocompatibility, biodegradability and toxicity in tissue engineering for several years, despite the disadvantages that it degrades rapidly and has a high swelling property (54,55). Therefore, a blend of both materials to make silk fibroin-chitosan (SFCS) may avoid the limitations of pure SF and CS. Furthermore, the blend has good mechanical properties and may be used as a scaffold material for the repair of SCIs (56,57).

In addition to SFCS scaffolds derived from natural components, injectable scaffolds are also particularly important for tissue engineering, as they are capable of filling the damaged site and may be delivered using a minimally invasive method (58). This type of scaffold also possesses the ability to mold to irregularly shaped damaged sites. Comolli *et al* (59) used a poly(N-isopropylacrylamide)-co-poly(ethylene glycol) (PNIPAAm-PEG) injection scaffold, which provided the sustained release of BDNF and neurotrophin-3 (NT-3) for up to four weeks; the constant secretion of these growth factors was identified to be a positive factor in functional recovery. However, the majority of these scaffolds require gelation (crosslinking) *in vivo,* which may result in complications, either from unreacted monomers or excess reactant (60).

Further to the two previously discussed types of scaffold, other scaffolds have been used successfully in the treatment of SCI. Kang *et al* (61) used poly (D,L-lactide-co-glycolide) to successfully treat transected spinal cords in rats; a certain degree of nerve regeneration and functional recovery was observed. A study by Liu *et al* (62) using nanofibrous collagen nerve conduits demonstrated that this type of scaffold is capable of promoting neural fiber growth following SCI, and is also capable of inhibiting glial scar hyperplasia. Zhu *et al* (63) used nanofibrous scaffolds as a drug delivery vehicle for the treatment of SCI in rats, and observed significant improvements in hindlimb function after three weeks. Teng *et al* (64) studied the use of a poly(lactic-co-glycolic acid) (PLGA) scaffold to treat SCI in rats. The authors identified that corticospinal tract fibers permeated the epicenter of the injury to the caudal cord and that local GAP-43 expression was increased, which lead them to hypothesize that PLGA increases the possibility of recovery following SCI. However, in contrast to the findings of Teng *et al,* a study by Du *et al* (65) demonstrated that a gelatin sponge is more suitable than a PLGA scaffold for transplantation into the spinal cord to promote the recovery of SCI.

In order to successfully use tissue engineering to repair SCI, the selection of a suitable scaffold is particularly important. Compared with a single component scaffold, a mixed scaffold (comprising several ingredients) may be more successful as it may minimize the disadvantages of the single component scaffold and provide a scaffold with increased functionality. Furthermore, compared with synthetic scaffolds, scaffolds prepared from natural components may be more advantageous, as the reaction of the immune system and the inflammatory reaction is reduced following implantation into the body.

## 4. Growth factor

Neurotrophic factors play an important role in functional recovery following SCI, as they protect neuronal cells from apoptosis and promote axonal regeneration (66). Neurotrophic factors may be divided into neurotrophins, ciliary neurotropic factor, the glial cell line-derived neurotrophic factor family and other growth factors or cytokines (67–69). The most frequently used neurotrophic factors are NGF, NT-3 and BDNF. NGF was discovered in 1950 and, as a core factor in the regulation of peripheral innervations, was found to have an effect on the CNS (70). Allen *et al* (71) demonstrated that NGF has a promising future as a therapeutic option for neurodegeneration. Weishaupt *et al* (72,73) extracted BDNF from a porcine brain and identified that it had a broad-spectrum effect on peripheral and central neurons, with the exception of the ciliary ganglion neurons, sensory neurons, hippocampal neurons, cerebellar neurons, motor neurons, cholinergic neurons of the basal forebrain and midbrain dopaminergic neurons. BDNF expresses its biological effects through the activation and binding of TrkB (74). Stokols *et al* (75) discovered that a BDNF-incorporated agarose scaffold implanted into the spinal cord of a rat resulted in the linear-fashioned growth of regenerating axons through the scaffold. NT-3, which may be generated by the cloning of a multifunctional NGF gene, not only maintains motor neurons, sympathetic neurons and dopaminergic neuron differentiation, but also maintains the survival of sympathetic and sensory neurons and promotes nerve outgrowth *in vitro* (76,77). To date, NT-3 is considered to be the only gene to promote the growth of the corticospinal tract (CST) following SCI. The biological effect of NT-3 is produced by the binding and activation of TrkC; NT-3 also has effects on TrkA and TrkB, but these are weak (78,79).

Neurotrophic factors may be applied by the following three methods: local injection, cerebrospinal fluid injection and gene modification (80). Of these methods, the most important is gene modification. Studies have shown that the direct injection of neurotrophins into the site of injury results in an improved functional recovery compared with that in a control group to which growth factor is not administered; however, due to several factors, such as concentration, time limitation, and a short half-life, this treatment was unable to fully meet the requirements for nerve regeneration (81,82). Cerebrospinal fluid injection has certain disadvantages, including the fact that it is not possible to restrict the location of the neurotrophic factor to the injury site, and recovery is less successful compared with that achieved using other methods. Therefore, an increasing number of researchers are focusing on gene modification (79).

There are two methods of applying gene therapy for the treatment of SCI (83–85): the direct transfer of the gene into the target cells in the human body, and cell-mediated gene therapy. The latter method, which requires the target gene to be transferred into an appropriate transplant cell, the selection of cells with a high level of gene expression, and the transplantation of the cells into the target tissue, is the most commonly used. Researchers have used transgenic technology to modify fibroblast cells, muscle cells and Schwann cells, which are subsequently transplanted into the injured area. The genetically modified cells may continue to express nutritional factor, the purpose of which is to promote nerve regeneration. Gene transfer vectors are divided into viral vectors and non-viral vectors (86). Viral vectors include adenoviruses, retroviruses and chronic viruses; retroviruses and chronic viruses are of particular interest as their transfer into the host genome may lead to long-term expression (87–89); Morizono and Chen (89) compared the efficiencies of three types of virus for the transfection of ADSCs, and observed that the highest transfection efficiency was achieved with a chronic virus. When an exogenous gene was transferred by chronic virus carriers into adipose stem cells, which were then induced *in vitro*, detection of the gene remained possible following osteogenic and adipogenic differentiation. Therefore, the combination of stem cells and chronic virus carriers is currently being studied (89). However, the clinical application may be simpler if growth factors are slowly released from scaffolds, rather than being delivered by gene transfer, using biomaterials that are capable of providing the protracted release of loaded proteins.

## 5. Conclusion

Tissue engineering is a promising method that may be used for the treatment of SCI. It involves three factors: the seed cell, the scaffold and a growth factor. For the repair strategy to be successful, the selection of an appropriate seed cell, scaffold and growth factor is required. Considering the seed cell, adult stem cells, particularly stem cells derived from adipose tissue, appear to be more suitable than fibroblasts, neuronal stem cells and ES cells. For the scaffold, scaffolds formed from natural components are more advantageous than scaffolds composed of artificial and synthetic materials. However, the blending of natural and synthetic materials may reduce the disadvantages of using synthetic material while also avoiding the disadvantages of using solely natural components. The growth factor is important as it enhances the repairing effect, particularly when the virus carrier used to transfect the stem cells enables the consistent expression of the growth factor gene. We hypothesize that the use of a combination of growth factors may be more effective than the use of a single growth factor. Therefore, the construction of a virus capable of carrying several genes together requires further study. In conclusion, although tissue engineering has a promising future for the treatment of SCI, extensive further studies are necessary for the successful treatment of SCI to be achieved.
